# Application of telemedicine system on the management of general patient in quarantine

**DOI:** 10.1038/s41598-023-37926-z

**Published:** 2023-07-27

**Authors:** Jiafa Lu, Xin Wang, Xiaolin Zeng, Wanjing Zhong, Wei Han

**Affiliations:** 1 Emergency Department of Shenzhen University General Hospital, Shenzhen, China; 2 Shenzhen Institute of Advanced Technology, Chinese Academy of Sciences, Shenzhen, China; 3 Department of Cardiology, Shenzhen University General Hospital, Shenzhen, China

**Keywords:** Disease prevention, Public health

## Abstract

To limit the epidemic of COVID-19, most countries and regions have adopted the policy of quarantine, providing an opportunity for the development of telemedicine. This study aims to develop a telemedicine system within a quarantined district and validate its effectiveness and safety in managing a variety of diseases within the population. Appling the private network and specialized set, telemedicine system and service process were constructed in the quarantine district. Based on the patients’ conditions, the staffs supplied kinds of medical service for the patients in the quarantine district. The basic characteristics and results of patients in the quarantine area who used telemedicine system during January to September 2022 were statistically analyzed. Within this period, 2410 cases were included in this study, among which, 1803 patients directly saw a doctor by the Internet hospital in the Internet hospital of telemedicine system, 607 patients used telemedicine system, 166 patients achieved referral to a specific hospital via telemedicine system, and 162 cases made further consultation, with no infection cases in the quarantine zone and no death cases. The six most occurred diseases were respiratory disease (20.6%), ophthalmology and otorhinolaryngology (12.9%), cardiovascular diseases (12.7%), digestive system disease (12.5%), dermatological diseases (10.6%), and metabolic and endocrine diseases (7.6%). The top three referred cases were obstetric diseases (19.3%), others (12.0%) and respiratory disease (10.2%). There were statistically significant differences between the diseases of the cases using telemedicine system with and without referral (*P* < 0.001). It is feasible, effective and efficient to construct and use telemedicine system in quarantine area. It is an approach to manage many patients by indirectly contact. With the solution of follow-up related problems and the application of novel technologies, telemedicine may usher in greater development.

## Introduction

The outbreak of COVID-19 had a broad impact on health, politics and economy. Medical systems around the world have been forced to adapt rapidly to telemedicine and digital innovation to reduce the spread of viruses^1^. The Ministry of the Interior and Transport of Japan plans to use 5G in the doctor to doctor remote diagnosis system during the epidemic^2^. During the epidemic, the US government eased various restrictions on telemedicine and provided additional funds for telemedicine^3^. Besides, China has carried out the benefit evaluation of telemedicine project application in Sichuan province as early as 2002^4^. Compared with developed countries, developing countries have higher requirements for telemedicine applications^5^. Due to medical facilities and personnel were relatively complete and sufficient in developed countries, while the resources were relatively scarce in developing countries. The construction of remote medical systems using existing network systems may be easier to complete than cultivating a large number of medical personnel in a short period of time. Because of the COVID-19 pandemic, the proceeding of telemedicine construction and usage were significantly accelerated^6^. Moreover, the pandemic also reminded us once again of the importance of using telemedicine to provide medical services, especially as a means to reduce the risk of cross-infection caused by close contact^7^.

Telemedicine uses modern communication, electronic and computer technologies to promote the remote collection, storage, processing, transmission and query of various medical information, so as to expand the scope of patients’ access to medical services by crossing geographical barriers. It is a discipline that provides clinical support for patients, thereby improving patient health. Many studies have proved that telemedicine can help avoid unnecessary long-distance transportation of patients, thus saving time and cost for patients and improving the quality of medical services^8^. Since telemedicine have been implemented for decades, more and more evidences are provided to prove the fact that telemedicine has the potential to improve the quality of patient care and reduce the hospital readmission rate^9^. At the same time, telemedicine provides the patients with more accesses to medical care, more time and cost efficiency, and improves patient satisfaction in a retrospective study^10^. Recently, telemedicine has been reported in the management of chronic diseases such as diabetes^10,11^, heart failure^12^, digestive tract diseases^13^, and hypertension^14^. Moreover, telemedicine also is applied in the prevention and control of COVID-19^15,16^. However, the abovementioned studies mainly focused on the feasibility or single disease, most of the previous studies were retrospective, and there are few reports on the management of multiple diseases for population. Hence, in this study, we try to verify the feasibility of telemedicine in managing multiple diseases by analyzing the management of multiple disease cases in quarantine area during the COVID-19 epidemic.

## Methods

### Subjects

**Figure 1:**
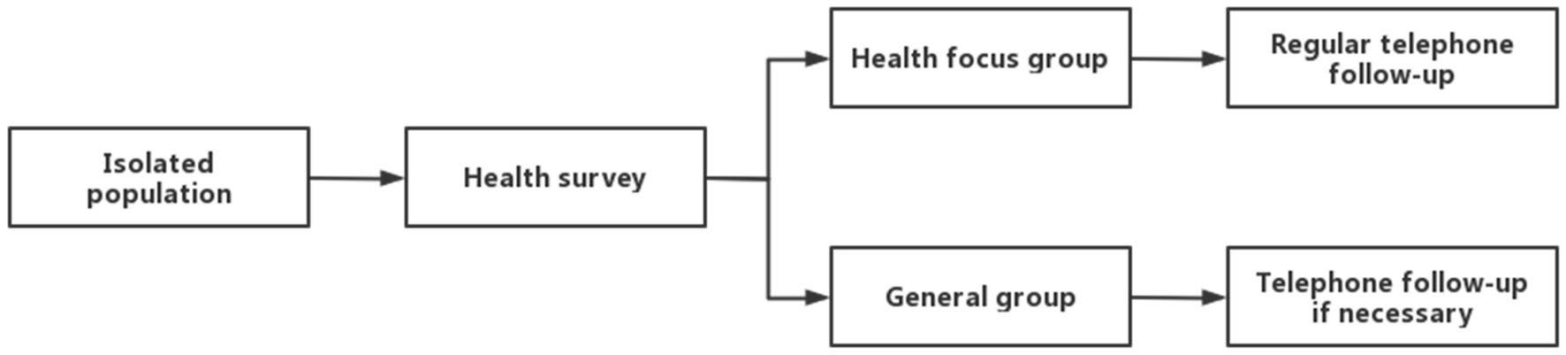
The workflow of classification for quarantine population.

In January 2022 and September 2022, the patients, including the quarantine personnel and staff in the area, who entered the designated quarantine area (a hotel district designated by the government and managed by professionals) in Shenzhen University General Hospital and went to see doctors through telemedicine system in the quarantine area were taken into consideration. The period is determined by the policy of quarantine of Shenzhen government. The personnel who mistakenly registered telemedicine system outside the quarantine or with no health needs were excluded. The informed consents had been obtained from all subjects involved, and this study was reviewed and approved by Shenzhen University General Hospital Research Professional Ethics Committee (KYLL-20230109A), and all methods were carried out in accordance with relevant guidelines and regulations. After entering the quarantine area, the personnel conducted health and psychological status survey and were divided into health focus group and general group accordingly. For the health focus group, regular online follow-up or on-site medical service were arranged, and for the patients who need regular monitoring of blood pressure, blood glucose, ECG, etc., medical staff were arranged to provide regular monitoring. The staff would inform the personnel in writing about the contact information for various needs, whether they were grouped in which group. The general population could ask for medical service of consultant at any time if they needed. The workflow of the classification for the quarantine personnel was shown in Fig. 1.

### Treatment process

**Figure 2:**
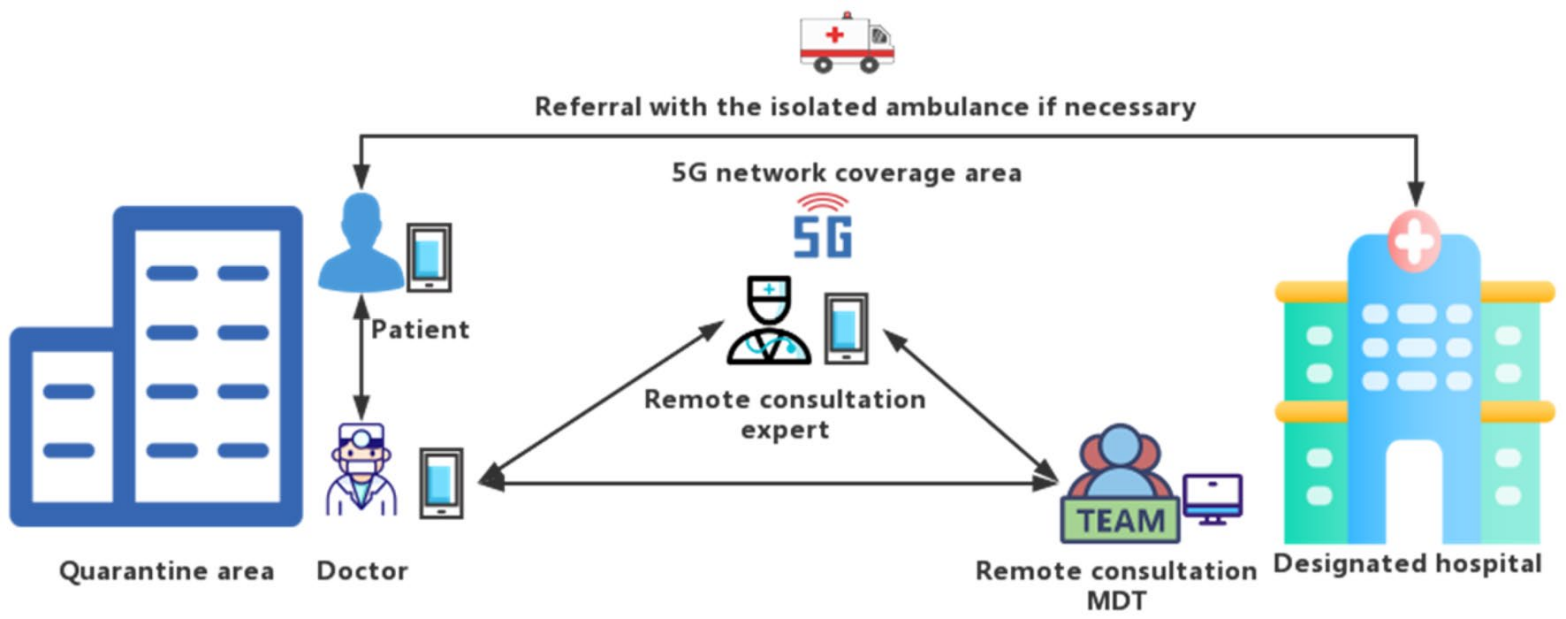
The workflow of medical consultation for isolated population.

**Figure 3:**
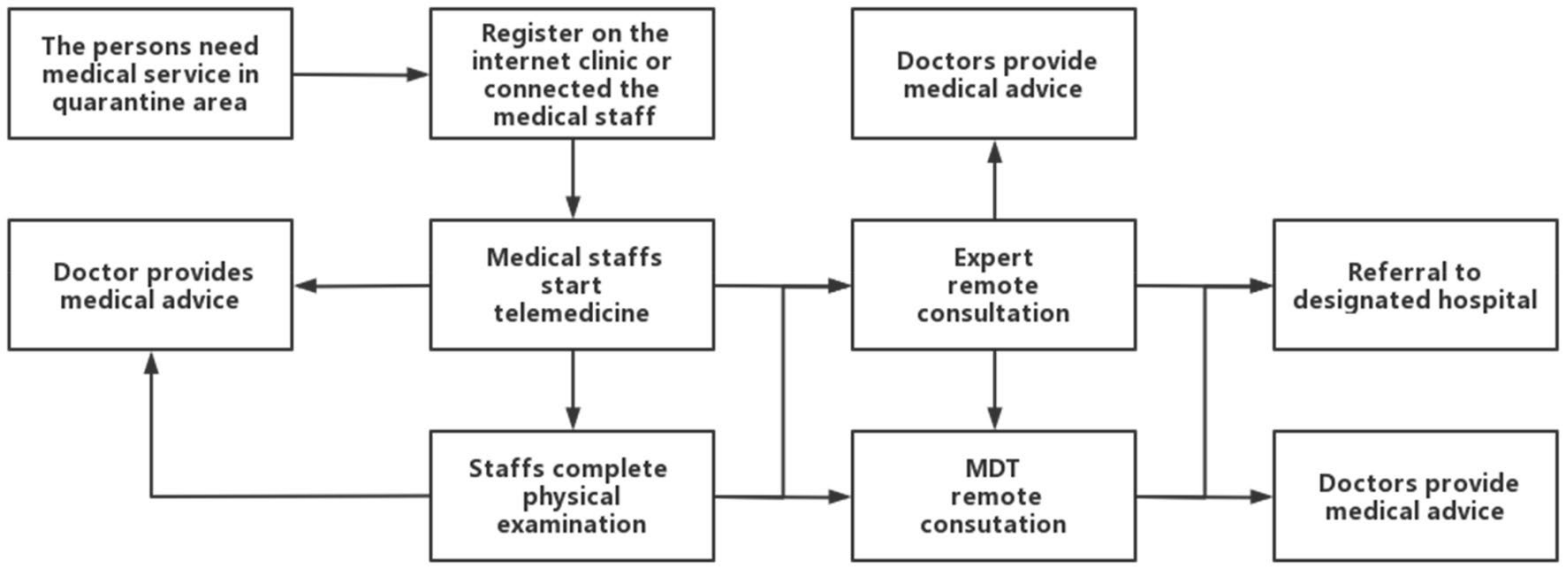
The frame of telemedicine system in the quarantine area.

When people in quarantine area needed to see a doctor, they could register online through the internet hospital established by the quarantine outpatient department. To those who did not use or were not familiar with the online registration in the internet hospital, they could directly call the medical staff whose phone number had been informed as aforementioned. After receiving the online registration from the internet hospital or the call from the patients, the medical staff would start telemedicine system. WeChat (Tencent Inc.) is the main communication method in telemedicine. For a few patients without WeChat, they were contacted by phone, or the WeChat of their caregivers, or of the staff in quarantine area for their telemedicine process. In order to keep information safety and unobstructed, the hotel provided special work computers, work phones, and dedicated work numbers and networks. Staffs were required to use specialized sets to communicate with patients. The doctors responsible for telemedicine include emergency doctors, general practitioners, psychologists, psychiatrists, and some specialists. Each building in quarantine area was equipped with special medical staff and independent dispensary which was equipped with basic drugs, first aid and inspection equipment. The first attending physician of telemedicine would directly give medical advice according to the patient’s condition, or contact the medical staff of the patient-located building for physical examination, either asked the experts in telemedicine to assist in the diagnosis according to the conditions. If necessary, multi-disciplinary treatment (MDT) could be applied to give medical advices according to the opinions of experts in telemedicine or MDT, as shown in Fig. 2. Telemedicine system in quarantine area built by 5G communication technology in this study mainly included three parts: Internet hospital and doctors, remote consultation system, and remote MDT, as shown in Fig. 3. Telemedicine system met the medical needs of personnel in the quarantine area, reduced direct contact, medical transport, the risk of infectious disease transmission, and ensured the medical safety of patients in the quarantine area. The construction of telemedicine system mainly relies on 5G network signals. The system has been passed the tests to verify the feasibility of this programme before the quarantine personnel and staff entered the quarantine area.

### Treatment of the diseases

Through telemedicine system, the first attending doctor or the medical team should give the preliminary diagnosis. If there were any needs for simple assist auxiliary examinations such as blood pressure, blood glucose, electrocardiogram (ECG), blood oxygen saturation, and so on, the first attending doctor could arrange the medical staff in the patient-located building to visit the patients for examination physically. For those who needed blood test, urine test, or computed tomography (CT), they could be arranged to finish these tests in the outpatient department in the quarantine area. Considering those who needed drug treatment, some of drugs that the pharmacy of the quarantine area had would be took and delivered to the quarantine room by specific person, while the drugs which were out of stock would be took from our hospital pharmacy or purchased outside. For the patients with medical diagnosis and treatment measures that the outpatient department could not meet, or with unknown causes, after the expert remote consultation or MDT remote consultation, for those could not be completed in the quarantine area, special medical transfer vehicles could be arranged to transfer to the designated hospital for diagnosis and treatment.

### Statistical analysis

The first attending doctor or medical team would first make the preliminary diagnosis according to the International Classification of Disease-10 (ICD-10). Considering the classification of departments in general hospitals in China and the preliminary diagnosis, the patients in quarantine were classified according to the type of diseases, mainly as 14 types: respiratory disease, cardiovascular diseases, digestive system disease, and so on. As for those with unclear causes or the diseases with patients’ number less than 1% of the total patients in this study, they were classified as others, as shown in Table 1. Online cases referred to those who registered directly by the Internet hospital and did not take part in remote expert assistance consultation, while those who participated remote consultation or remote MDT were recognized as remote expert assistance consultation cases. Referral patients meant those who were referred after expert or MDT consultation. Patients who had a return visit within 1 week for the same reason or disease were defined as revisiting patients.

**Table 1:** Basic clinical characteristics of the patients and the consultation results.

Clinical characteristics	
Age, y	35.5 ± 14.8
Male (%)	1338 (55.5%)
Respiratory disease	497 (20.6%)
Ophthalmology and otorhinolaryngology	312 (12.9%)
Cardiovascular diseases	305 (12.7%)
Digestive system disease	302 (12.5%)
Dermatological diseases	256 (10.6%)
Metabolic and endocrine diseases	183 (7.6%)
Psychological or psychiatric disorders	154 (6.4%)
Injury	122 (5.1%)
Nervous system diseases	77 (3.2%)
Others	63 (2.6%)
Urinary system diseases	55 (2.3%)
Gynecological diseases	45 (1.9%)
Obstetric diseases	39 (1.6%)
Consultation results	
Online consultation only	1803 (74.8%)
Used remote expert assistance consultation	607 (25.2%)
Transfered after remote expert assistance consultation	166 (6.9%)
Further consultation	162 (6.7%)

The ages of patients were measurement data, represented by mean ± SD. Enumeration data such as patient's sex and disease type were expressed by frequency (percentage). The comparison of two sample rates was performed by chi square test. If there were rows or columns with too small theoretical frequency, the rows or columns with similar properties were combined according to clinical experience. SAS 9.4 (SAS Institute Inc, USA) was used for statistical analysis and the difference was considered as statistically significant with *P* < 0.05.

### Ethical approval and consent to participate

This project has been approved by Shenzhen University General Hospital Research Professional Ethics Committe (KYLL-20230109A). The informed consents had been obtained from all subjects involved.

## Results

### Basic information about patients of using telemedicine systems

**Figure 4:**
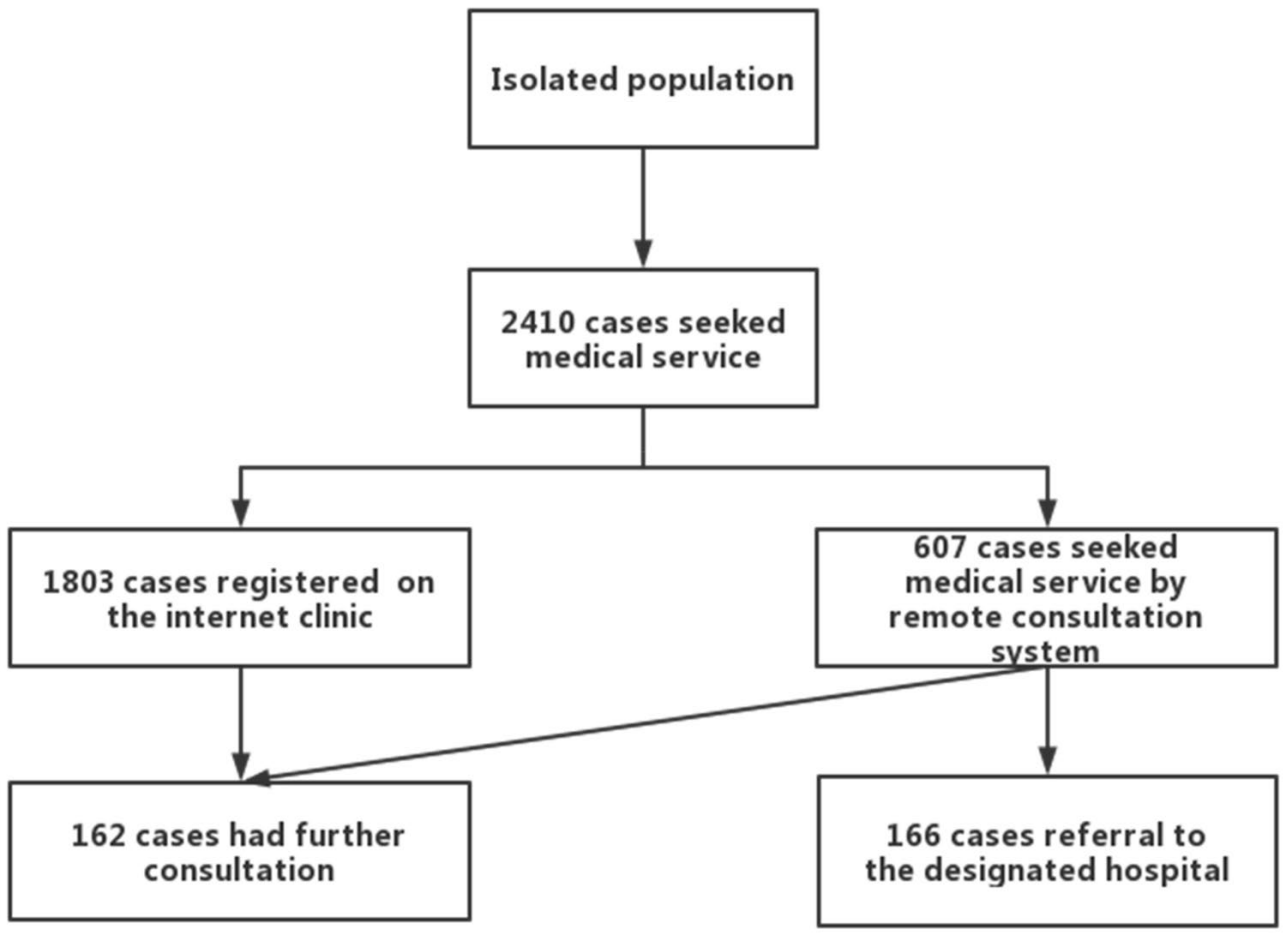
The result of the population used telemedicine system in quarantine area.

During this study, 2410 quarantined people used telemedicine system. Among them, 1803 patients directly went to the Internet hospital in telemedicine system, 607 patients used the remote expert consultation system after consulting the doctor in the quarantine area with telemedicine system, 166 patients were transferred to the designated hospital after the remote expert assistance telemedicine or MDT, and 162 patients went for further (revisiting) consultation (as shown in Fig. 4 and Table 1).

### Basic clinical characteristics of patients using telemedicine systems

Among 2410 telemedicine-used patients, 1338 were male, accounting for 55.5%, aged from 1 month old to 91 years old, with an average age of 35.5 ± 14.8 years old, and 74.0% were under 45 years old. Sorting from most to least, the diseases were respiratory disease (20.6%), Ophthalmology and Otorhinolaryngology (12.9%), cardiovascular diseases (12.7%), digestive system disease (12.5%), dermatological diseases (10.6%), Metabolic and endocrine diseases (7.6%), psychological or psychiatric disorders (6.4%), injury (5.1%), nervous system diseases (3.2%), others including tumor, chronic infectious diseases, nutritional deficiency, unknown causes, et al. (2.6%), urinary system diseases (2.3%), gynecological diseases (1.9%), obstetric diseases (1.6%). Among them, 74.8% of patients only used telemedicine system, 25.2% used remote expert assistance consultation system or MDT consultation system after, 6.9% of patients transferred to designated hospitals after telemedicine consultation, and 6.7% of patients returned for consultation. In the quarantine area, there were no serious medical accidents, no death cases, and no infection, the details were shown in Table 1.

### Comparison of referrals and non-referrals in patients who used the teleconsultation system

As shown in Table 2, among 607 patients who used the remote consultation system, the patients suffered respiratory disease and cardiovascular diseases used most, accounting for 16.0% and 12.9% respectively. The first several diseases requesting remote consultation were not completely consistent with the order of diseases requesting medical services in the entire quarantine area. 441 cases were not referred, accounting for 72.7%; 166 cases were referred, accounting for 27.3%, of which 55.4% were women. The order of disease types of patients not being referred is consistent with that of patients seeking remote consultation. The patients with obstetric diseases were the most frequently referred, accounting for 19.3% of the transferred patients, followed by patients with other diseases of unknown causes. There were no dermatological diseases and psychological or psychiatric disorders patients referred. The difference of disease category rate between referral group and non-referral group was statistically significant (*P* < 0.001).

**Table 2:** Basic clinical characteristics of the patients with remote consultation cases.

Clinical characteristics	Remote consultation cases	No referral group*	Referral group*
Age, y	36.0 ± 15.7	37.3 ± 15.7	32.6 ± 15.0
Male (%)	322 (53.3%)	248 (56.2%)	74 (44.6%)
Respiratory disease	97 (16.0%)	80 (18.1%)	17 (10.2%)
Cardiovascular diseases	78 (12.9%)	68 (15.4%)	10 (6.0%)
Digestive system disease	59 (9.7%)	46 (10.4%)	13 (7.8%)
Metabolic and endocrine diseases	54 (8.9%)	45 (10.2%)	9 (5.4%)
Ophthalmology and Otorhinolaryngology	53 (8.7%)	39 (8.8%)	14 (8.4%)
Psychological or psychiatric disorders	52 (8.6%)	52 (11.8%)	0 (0.0%)
Injury	37 (6.1%)	21 (4.8%)	16 (9.6%)
Obstetric diseases	36 (5.9%)	4 (0.9%)	32 (19.3%)
Dermatological diseases	36 (5.9%)	36 (8.2%)	0 (0.0%)
Others	27 (4.4%)	7 (1.6%)	20 (12.0%)
Nervous system diseases	25 (4.1%)	22 (5.0%)	3 (1.8%)
Gynecological diseases	22 (3.6%)	7 (1.6%)	15 (9.0%)
Urinary system diseases	22 (3.6%)	11 (2.5%)	11 (6.6%)
Tumour	9 (1.5%)	3 (0.7%)	6 (3.6%)
Total	607 (100.0%)	441 (100.0%)	166 (100.0%)

* χ^2^ = 135.581, *P* < 0.001.

## Discussion

Telemedicine can also be briefly referred to as the application of information and telecommunications technology in medicine, not only for remote monitoring or diagnosis of patients, but also for remote education and remote consulting services for medical staff and patients^5^. According to a trend report of Langone Health of NYU, in the early days of COVID-19, the number of patients using telemedicine increased by 683%^17^. In the early days of the outbreak of the epidemic, SUGH opened an internet hospital to provide free telemedicine consulting services for citizens during the epidemic, provide necessary medical information services and medical support for the public with health consulting needs, reduce social panic, promote safe social isolation, improve the public's self-protection ability^18^, and also provide our team with telemedicine experience in implementing this medical security task.

After the outbreak of the epidemic, the Chinese government implemented stricter social isolation policies, such as social isolation and community containment, as the main strategy to control the epidemic of COVID-19^19^. A simulation study showed that institution based isolation is more efficient than family based isolation in reducing the number of new cases of COVID-19^20^. Telemedicine is booming worldwide and has become an indispensable resource for improving the health management of quarantined patients^14^. The Shenzhen government also mainly adopted the policy of centralized quarantine, supplemented by home isolation, to isolate the close contacts of COVID-19, and provided all kinds of security including living and medical care for the isolated personnel. In this way, the outbreak of the epidemic was quickly controlled under the condition of multiple outbreaks.

This study mainly verified the feasibility and safety of telemedicine in managing multiple diseases in the quarantine area during the epidemic. Through analysis, we found that the average age of 2410 patients was 35.5 years old, which was close to the average age of 32.5 years old of Shenzhen’s permanent population reported by the media^21^. Among the diseases, the top ones were respiratory disease, ophthalmology and otorhinolaryngology, cardiovascular diseases, digestive system disease, dermatological diseases, metabolic and endocrine diseases, which are inconsistent with the order of the top diseases reported in the outpatient of other regions. The top diseases of patients in common communities were mainly respiratory infection, hypertension, diabetes and other diseases, excluding ophthalmology, otorololygeology and dermatological diseases, which might be contributed to the fact that the majority of patients in common community were middle-aged and elderly^22,23^. In this study, most patients were under 45 years old. Long term use of electronic products was likely to cause discomfort to the eyes. Also, in a relatively closed environment in the isolation area, it might be more likely to cause problems such as eye, mouth and skin discomfort due to diet, environment and other factors. However, no matter in the common community or in the isolated community of our study, respiratory disease was one of our common diseases.

Through the establishment of a telemedicine system in isolated communities, the medical service scheme, which focuses on online medical treatment and supplemented by offline medical treatment, provides various medical services for people in isolated communities. In our study, only 166 of 2410 patients were transferred to designated hospitals after remote consultation, accounting for 6.9% of all patients, which greatly reduced the need for patients in isolated communities to go out to see doctors and reduced the risk of community transmission of infectious diseases. Like other studies, our scheme showed advantages in saving time and medical resources. During the epidemic, telemedicine has become an increasingly valuable patient service resource^24^. In our research, we also realized that the total evaluation time of telemedicine group was often longer than that of our offline routine group, which might be because doctors spend more time communicating with patients and providing explanations to patients^25^. However, because the doctors involved in the project had different experience in telemedicine, inconsistent complexity of the diseases, and inconsistent understanding ability of the patients, we did not make statistics in this regard. This also reflected the difference between telemedicine and the traditional offline medical treatment model. Telemedicine pays more attention to communication skills between doctors and patients.

Among all the patients we received, 607 patients in total used the remote consultation system. After the remote expert consultation, 441 patients solved their medical problems. 166 patients were transferred to designated hospitals for treatment. There was a significant difference in disease types between the referral patients and the non-referral patients. Among the referral patients, the patients who need obstetric examination were the most, followed by those who needed more auxiliary examinations for diagnosis due to unknown causes. This was mainly because there were no obstetric related examination equipment and obstetricians, and some patients had complex conditions that could not be diagnosed or exceeded the scope of medical resources provided in the quarantine area. During the whole study period, there were no deaths or infections in the quarantine area. In a medical management of the isolation area in Singapore, they also adopted telemedicine system. 136 patients were transferred to the hospital, and there were no deaths in the isolation area and no infections among medical staff, which also proved the feasibility of telemedicine in managing patients in the isolation area^16^.

Therefore, telemedicine is considered as a safe and feasible solution to manage patients in isolation areas, and the success of telemedicine may provide useful reference for other regions of the world. It was reported that 76% of hospitals in the United States contact patients through some form of telemedicine. Doctors in radiology (39.5%), psychiatry (27.8%) and cardiology (24.1%) were the most common users of telemedicine^9^. telemedicine network in Sichuan, China, combines the newly established 5G services, smart phone applications and the existing telemedicine system, which has also been proved to be feasible, acceptable and effective in western China, and has significantly improved the medical results^26^.

The technology of telemedicine is matured, but the real challenge is to change the habits of doctors and patients, and better supervision is needed^27^. The realization of telemedicine benefits has nothing to do with the technology itself, but more depends on the organization and its implementation capability^28^. Lack of funds is considered to be one of the reasons for the slow popularization of telemedicine. Others believed that telemedicine training was limited and doctors needed to learn new methods. Doctors' unwillingness to adopt telemedicine also limited the rapid development of telemedicine^7^. As mentioned earlier, telemedicine is not exactly the same as traditional offline medicine. In addition, due to the lack of personal care in telemedicine, medical staff may express concern about this^29^. The challenges facing telemedicine include the lack of technical infrastructure in some regions, financial constraints and health system priorities conflict, medical and legal issues^30,31^, reimbursement, and the interests of medical payer and insurance provider^9^, as well as the lack of mutual recognition of licenses between regions, popularization of necessary technologies, concerns about medical liability issues and limited medical cases^32^. Although telemedicine cannot solve all problems, it is very suitable for the situation where the infrastructure is intact and the clinicians can execute it^33^. With the development of remote diagnosis and wearable sensors, telemedicine has been effectively used for the prevention and treatment of COVID-19^34^, and will probably be used for the prevention and treatment of more kinds of diseases.

This project is a public welfare project supported by government funds. The construction cost and maintenance cost of telemedicine system, as well as the patient information security and the human resource cost of the professional and technical personnel involved in telemedicine were not discussed in this project. In order to ensure the information security of patients, all staffs in the quarantine district have signed a confidentiality agreement and were required to use of private networks, special computers, mobile phones and other specialized sets. Due to the scarcity of human resources, we had an insufficient personnel to the work of feedback, and the system does not include feedback from patients, so there was a lack of objective evaluation of whether it had provided good service. This was also a part of the limitations of this study. Future research should not only consider the benefits of telemedicine to patients, but also consider the information security and overall operating costs of telemedicine, and compare it with traditional offline medical systems to more comprehensively evaluate the application value of telemedicine.

## Conclusion

It is proved that telemedicine is feasible and safe in multi-diseases management in quarantine area, which save times for the patients to go out to see a doctor, reduce the financial costs and the risk of infectious disease transmission. However, the promotion of telemedicine still faces a lot of restrictions, such as financial, legal and political support, information safety, operating costs, and the participation of medical staff. Nevertheless, with the aforementioned problems solved and the development of the wearable devices, telemedicine is likely to usher in greater development.

## Data Availability

The datasets generated and/or analysed during the current study are not publicly available due to the policy of privacy protection but are available from the corresponding author on reasonable request.
